# Monitoring the Introduction of Pneumococcal Conjugate Vaccines into West Africa: Design and Implementation of a Population-Based Surveillance System

**DOI:** 10.1371/journal.pmed.1001161

**Published:** 2012-01-17

**Authors:** Grant A. Mackenzie, Ian D. Plumb, Sana Sambou, Debasish Saha, Uchendu Uchendu, Bolanle Akinsola, Usman N. Ikumapayi, Ignatius Baldeh, Effua Usuf, Kebba Touray, Momodou Jasseh, Stephen R. C. Howie, Andre Wattiaux, Ellen Lee, Maria Deloria Knoll, Orin S. Levine, Brian M. Greenwood, Richard A. Adegbola, Philip C. Hill

**Affiliations:** 1 MRC Unit, Fajara and Basse stations, The Gambia, West Africa; 2 Disease Control Programme, Department of State for Health, Government of The Gambia, The Quadrangle, Banjul, The Gambia, West Africa; 3 PneumoADIP, International Vaccine Access Centre, Johns Hopkins Bloomberg School of Public Health, Baltimore, Maryland, United States of America; 4 Infectious & Tropical Diseases Department, London School of Hygiene and Tropical Medicine, London, United Kingdom; 5 Global Health, Infectious Diseases Division, Bill & Melinda Gates Foundation, Seattle, Washington, United States of America; 6 Centre for International Health, Faculty of Medicine, University of Otago, Dunedin, New Zealand

## Abstract

Philip Campbell Hill and colleagues describe how they set up a population-based surveillance system to assess the impact of pneumococcal conjugate vaccines on invasive pneumococcal disease (IPD) and radiological pneumonia in children in The Gambia.

**Summary Points**

- Routine use of pneumococcal conjugate vaccines (PCVs) in developing countries is expected to lead to a significant reduction in childhood deaths. However, PCVs have been associated with replacement disease with non-vaccine serotypes.
- We established a population-based surveillance system to document the direct and indirect impact of PCVs on the incidence of invasive pneumococcal disease (IPD) and radiological pneumonia in those aged 2 months and older in The Gambia, and to monitor changes in serotype-specific IPD.
- Here we describe how this surveillance system was set up and is being operated as a partnership between the Medical Research Council Unit and the Gambian Government.
- This surveillance system is expected to provide crucial information for immunisation policy and serves as a potential model for those introducing routine PCV vaccination in diverse settings.

## Introduction and Rationale

The introduction of routine immunisation with pneumococcal conjugate vaccines (PCVs) in developing countries is expected to significantly reduce childhood deaths [1]. The 7-valent pneumococcal conjugate vaccine (PCV-7), containing serotypes 4, 6B, 9V, 14, 18C, 19F, and 23F, is highly efficacious against invasive pneumococcal disease (IPD) of vaccine serotype, plus serotype 6A [2],[3]. Routine immunisation with PCV-7 has led to dramatic decreases in IPD due to pneumococci of vaccine serotype, but variable increases in IPD due to pneumococci of non-vaccine serotype [4]–[6]. Therefore, it is essential to monitor the introduction of PCVs in different settings. Demonstrating the impact of PCVs will be crucial for ongoing immunisation policy.

In 2009, the Government of The Gambia introduced routine PCV-7 vaccination into the national Expanded Programme of Immunisation (EPI) with support from the GAVI Alliance. Three doses of PCV-7 are given at 2, 3, and 4 months of age. A limited catch-up campaign gave at least two doses of vaccine to approximately 50% of children aged 2–11 months and one dose of vaccine to approximately 10% of children aged 12–23 months. PCV-13 replaced PCV-7 in April 2011 without a catch-up campaign.

The importance of pneumococcal disease in The Gambia was indicated by a 16% reduction in all-cause mortality in children 2–29 months of age in a trial of PCV-9 conducted in Upper and Central River Regions [7]. There was a 71% reduction in IPD of vaccine serotype and a 35% reduction in radiological pneumonia. The incidence of IPD and radiological pneumonia in the placebo arm was 390 per 100,000 person years and 37 per 1,000 person years, respectively. The incidence of IPD in this part of The Gambia was estimated previously to be 554 and 240 per 100,000 person years in under 1 and under 5 year-old children, respectively [7]. Neither the burden of IPD, nor the extent of the indirect (herd) effect of PCVs, are known in older children and adults in The Gambia, although PCV-7 vaccination of Gambian infants has recently been shown to reduce carriage of vaccine serotypes in older subjects [8].

Serotype coverage of IPD in The Gambia with PCV-7 and PCV-13 is approximately 30% and 70%, respectively [9]. Consequently, there is a large reservoir of pneumococci of non-vaccine serotypes available for replacement [10]. We developed a surveillance system to monitor the incidence of vaccine and non-vaccine type IPD and radiological pneumonia in those aged 2 months and older, before and after the introduction of PCVs. The data, when available, will provide key information to support PCV immunisation policy in The Gambia and elsewhere in Africa. Here we describe how this surveillance system was set up and is being operated.

## Purpose

The surveillance system monitors two key outcome measures before and after the introduction of PCVs: (1) the incidence of IPD due to vaccine and non-vaccine serotypes and (2) the incidence of radiological pneumonia. Secondary aims are to monitor: (1) pneumococcal antimicrobial resistance and (2) child mortality.

## Population under Surveillance

### Surveillance Site

**Figure 1:**
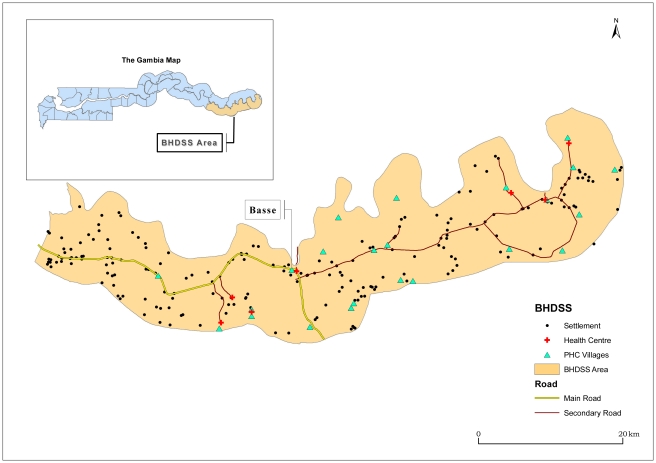
Map of catchment area for the surveillance system in The Gambia, including settlements, primary health care (PHC), and other health facilities.

The Gambia is a small West African country of 1.5 million people. The surveillance system was established in the Upper River Region and is integrated with the local health system. The only major health centre in the Upper River Region is in the town of Basse, serving a largely rural population of approximately 191,000 people on both banks of the river Gambia. The Basse Health and Demographic Surveillance System (BHDSS) is restricted to the population on the south bank of the river (Figure 1). Five peripheral clinics in the BHDSS area provide inpatient and outpatient care and refer patients to Basse health centre.

### Demographic Surveillance

To provide precise estimates of the population denominator, demographic surveillance commenced in July 2007 with visits to each household every 4 months. The total population initially enumerated was 136,387. All deaths, births, in- and out-migrations, pregnancies and marriages, and the vaccination status of the children, are recorded during visits. Verbal autopsies are conducted using questionnaires designed by the INDEPTH Network (http://www.indepth-network.org). The Global Positioning System coordinates of all households have been recorded.

### Sample Size Considerations

We estimated that the surveillance system would identify approximately 75 cases of IPD in children under 5 years of age per year [7], 45 of whom would be under 2 years of age, prior to the introduction of PCV-7 [3]. Using incidence data from the placebo arm of the PCV-9 trial in The Gambia, we calculated that we would have approximately 85% power to detect a 50% decrease in vaccine type IPD in under 2-year-old children over 2 years of before and after surveillance, at the 5% level of significance. We calculated that we would have over 90% power to detect a 20% reduction in radiological pneumonia in children under 2 years old and 60%–70% power to detect a 40% increase in non-vaccine type IPD. Increased power of the study has resulted from extension beyond 2 years of follow-up and the growth in the population under surveillance to over 160,000 in 2011. In addition, the introduction of the higher valency PCV-13 (containing the additional serotypes 1, 3, 5, 6A, 7F, and 19A) in mid-2011, should further reduce vaccine type IPD and radiological pneumonia. Sample size considerations assumed vaccination coverage of over 85%.

### Case Definitions and Screening Criteria

#### Case definitions

Case definitions were developed to ensure international comparability while being measurable locally (Table 1). IPD includes any case of clinically suspected pneumonia, meningitis, or septicaemia from whom *S. pneumoniae* is isolated from a normally sterile site. The case definition for radiological pneumonia is aligned to the World Health Organization (WHO) consensus definition, as used previously [3],[11].

**Table 1:** Case definitions for pneumonia, meningitis, and septicaemia.

Syndrome	Case Definition
**Bacterial pneumonia**	A suspected case of pneumonia with confirmed aetiology by isolation of bacteria from a normally sterile site (e.g., blood, pleural fluid, or lung aspirate)
**Radiological pneumonia**	A suspected case of pneumonia with changes on chest X-ray that meet WHO standard criteria for end-point consolidation
**Meningitis**	A suspected case of meningitis with confirmed bacterial aetiology from CSF or blood
**Septicaemia**	A suspected case of pneumonia, meningitis, or septicaemia with confirmed aetiology by isolation of pathogenic bacteria from the blood

#### Screening criteria for nursing assessment

**Figure 2:**
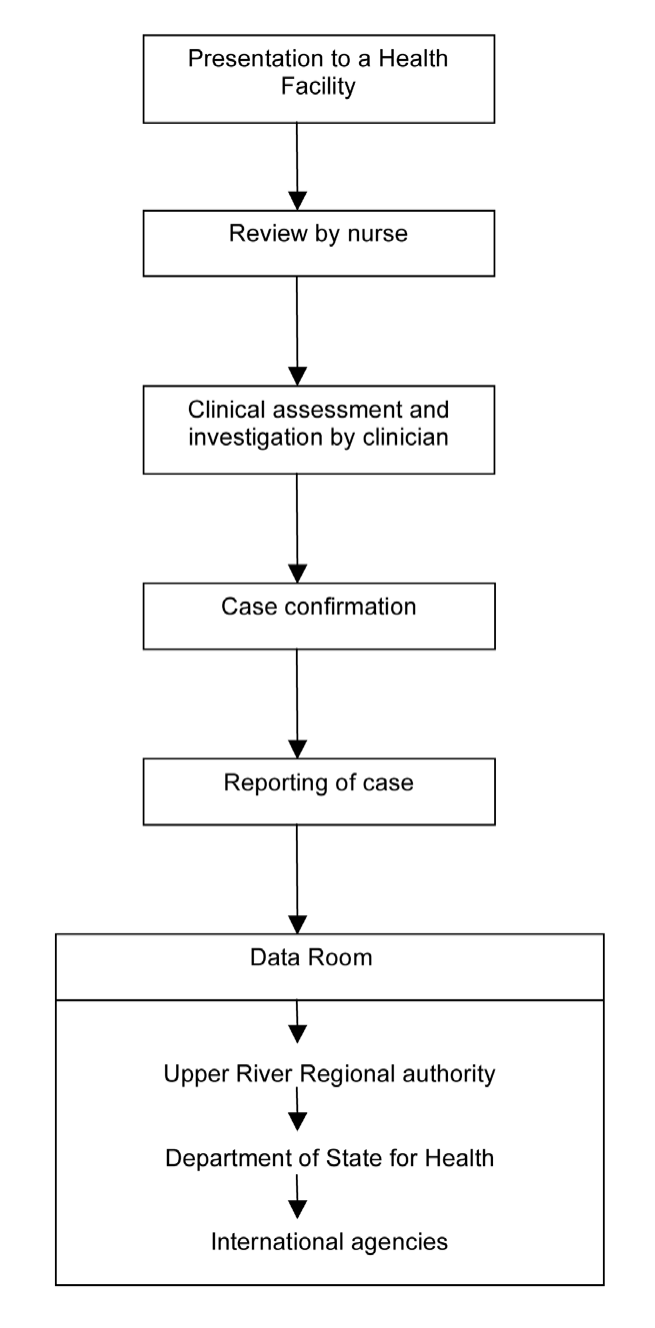
Flow chart for the Gambian pneumococcal surveillance system.

Figure 2 illustrates the flow from presentation of a case to formal reporting of the clinical and laboratory data. All individuals in the BHDSS population who present to health facilities at any time of day are evaluated by a dedicated surveillance system nurse to determine if referral to a surveillance system clinician at Basse is indicated. The nurses also conduct daily rounds of all inpatients to identify any cases missed at outpatient screening or who have developed criteria for referral. Criteria for referral are presented in Table 2, and these took into consideration the capabilities of local nurses. Separate criteria were developed for patients aged 2–59 months and for those 5 years and older. Patients who do not meet referral criteria proceed to evaluation and treatment by other health facility staff.

**Table 2:** Criteria developed for nurses to identify patients who should be referred to a clinician for assessment of suspected pneumonia, meningitis, or septicaemia.

Criteria Definition^a^	
≥2 Months and <5 Years	≥5 Years
History of cough or difficulty breathing, AND raised respiratory rate for age^b^	History of cough and difficulty breathing
Axillary temperature of at least 38°C, or less than 36°C in a patient admitted or being admitted	History of cough and pleuritic chest pain
History of convulsion	History of cough and supraclavicular/sternal recession or nasal flaring
Impaired consciousness^c^	History of productive cough and fever
Bulging fontanelle	History of rigors
Stiff neck	History of seizure
Prostration^d^	Impaired consciousness^c^
Lower chest wall indrawing, nasal flaring, or grunting	Altered mental state
Oxygen saturation less than 92%	Axillary temperature of at least 38°C or less than 36°C in a patient admitted or being admitted or
Weight below −3 z-score for age	Photophobia
Local musculoskeletal swelling or tenderness	Neck stiffness
Any child with suspected meningitis	Local musculoskeletal swelling or tenderness
—	Any patient with suspected meningitis

a To be referred for further assessment if one or more of the following are present for 14 days or less.

b Raised respiratory rate for age is defined as greater than 50 breaths per minute for children at least 2 months but less than 12 months, and as greater than 40 breaths per minute for children at least 12 months but less than 60 months.

c Impaired consciousness is defined as V, P, or U on the AVPU score, where A is if the patient is alert, V if responsive to verbal stimulus, P if responsive to pain stimulus, and U if unresponsive.

d Prostration is defined as inability to drink or breast feed, or to remain sitting in a child otherwise able to sit.

#### Screening criteria for clinical assessment

Patients referred by the nurses are evaluated by clinicians using clinical criteria for suspected pneumonia, meningitis, and septicaemia (Table 3). These were derived from WHO definitions [12] and previous studies [3],[7]. In addition, cough and/or difficulty breathing with an oxygen saturation <92% on breathing air was included as a criterion for suspected pneumonia [13]. We adapted criteria for lumbar puncture from a study of adults in Malawi [14]. For children under 5 years of age, severe malnutrition was included as an indication for blood culture [15].

**Table 3:** Clinical criteria for suspected pneumonia, meningitis, and septicaemia.

Suspected Condition	Criteria Definition			
	≥2 Months and <5 Years		≥5 Years	
**Suspected pneumonia**	Pneumonia is suspected if there is a history of cough or difficulty breathing of less than 14 days' duration, accompanied by one or more of:	1. Raised respiratory rate for age^a^	Pneumonia is suspected in patients presenting with an illness of 14 days' duration or less, if two or more of the following are present:	1. Cough
		2. Lower chest wall indrawing, nasal flaring, or grunting		2. Haemoptysis
		3. Oxygen saturation less than 92%		3. Pleuritic chest pain
		4. Focal chest signs (dull percussion note, coarse crackles, bronchial breathing)		4. Breathlessness
		—		5. Axillary temperature ≥38°C
**Suspected meningitis**	Meningitis is suspected if the patient is clinically unwell and if any of the following are present:	1. Neck stiffness	Meningitis is suspected if the patient is clinically unwell and if two or more of the following are present:	1. Axillary temperature ≥38°C
		2. Impaired consciousness^b^		2. Meningism (neck stiffness and/or photophobia)
		3. Prostration^c^		3. Altered mental state (Glasgow Coma Score <4)
		4. History of convulsion		—
		5. Bulging fontanelle		—
**Suspected septicaemia**	Septicaemia is suspected if one or more of the following is present:	1. Clinician diagnosis of focal sepsis (including but not limited to: septic arthritis, osteomyelitis, endocarditis, peritonitis, liver abscess, soft tissue abscess, cellulitis)	Septicaemia is suspected if one or more of the following is present:	1. Clinician diagnosis of focal sepsis (including but not limited to: septic arthritis, osteomyelitis, endocarditis, peritonitis, liver abscess, soft tissue abscess, cellulitis)
		2. Axillary temperature is <36°C or ≥38°C and no obvious cause of fever		2. Axillary temperature is <36°C or ≥38°C and no obvious cause of fever
		3. For a patient admitted, or being admitted, the clinical impression is of severe malnutrition^d^.		3. History of rigors

a Raised respiratory rate for age is defined as greater than 50 breaths per minute for children at least 2 months but less than 12 months, and as greater than 40 breaths per minute for children at least 12 months but less than 60 months.

b Impaired consciousness is defined as V, P, or U on the AVPU score, where A is if the patient is alert, V if responsive to verbal stimulus, P if responsive to pain stimulus, and U if unresponsive.

c Prostration is defined as inability to drink or breast feed, or to remain in a seated position in a child otherwise able to do so.

d Severe malnutrition is defined according to the WHO definition.

We initially adopted a temperature criterion of <36°C or ≥38°C for referral as a suspected case of bacteraemia, following a previous study in Gambian children [16]. However, because this criterion overwhelmed the surveillance capacity during piloting, with little added yield of cases of IPD, it was applied to inpatients only. Similarly, “excessive crying” was omitted because of a low yield and a large number of inappropriate referrals.

These criteria are applied in the presence of sound clinical judgement. In particular clinicians are trained that clinical judgement plays an important role in identifying patients who might have meningitis (Table 3). Clinical assessment results in diagnoses of suspected pneumonia, meningitis, septicaemia, or combinations of the three. Standardised investigations are performed on the basis of the surveillance diagnosis. Most specimens and all X-rays are taken at Basse Health Centre. If necessary, blood cultures are collected at peripheral clinics prior to antibiotic administration and transport of the patient. Lung aspiration is undertaken on children with dense and accessible peripheral consolidation on chest X-ray after informed consent.

### Implementation of the Surveillance System

#### Situational analysis

We conducted pre-surveillance site evaluations of clinical and laboratory facilities, including evaluation of patient referral and investigation, transport, electricity supply, staff expertise and training, and existing clinic and laboratory infrastructure. We reviewed patient flows and the systems employed for their transport between facilities.

#### Project management

**Figure 3:**
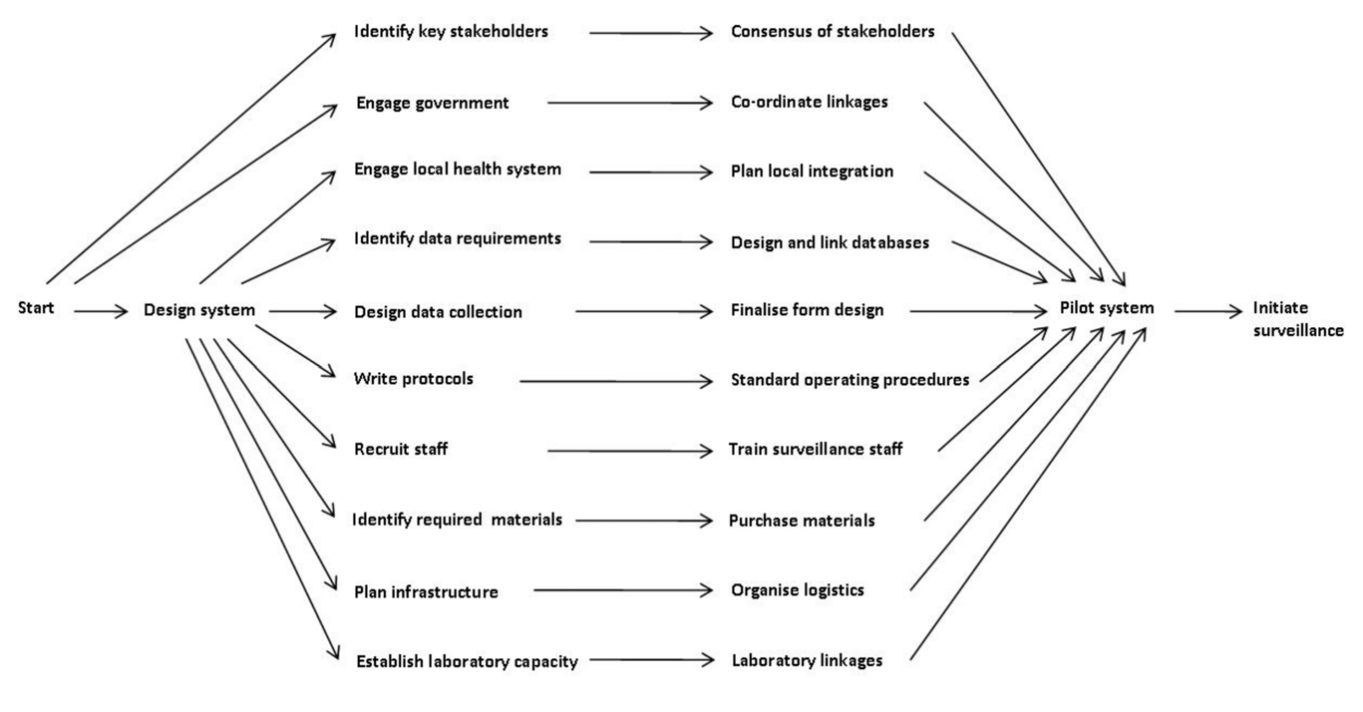
Project management for the establishment of the Gambian pneumococcal surveillance system: major dependency relationships.

We used a project management framework to set up the surveillance system. Figure 3 shows each line of activity as a series of dependency relationships, which were then prioritised according to estimated time to completion. We aimed to complement routine assessment and management, while strengthening existing clinical and laboratory services. We organised meetings at central, regional, and local level, and developed plans in collaboration with staff at clinics, the health centre, and the regional health office. We explained the nature and purpose of the project employing oral and visual presentations at community events and answered participants' questions.

#### Facilities

A system was established to transport patients referred to Basse Health Centre for clinical evaluation and a separate space was established at the health centre for the assessment. A digital X-ray system was established for pneumonia surveillance. The MRC Basse laboratory performs all microbiological analyses. The MRC laboratory on the coast performs serotyping and is a WHO reference site for pneumococcal bacteriology and serotyping.

#### Staff and staff training

On average, four clinicians (all based in Basse) and 16 nurses (11 based in Basse) are employed on the surveillance team, along with auxiliary staff and a medically trained epidemiologist. Surveillance staff are located at each health facility serving the BHDSS population. A radiographer, trained for previous studies, was retained and employed to work on the surveillance system. Training was given to project staff on assessment of patients using surveillance criteria through oral and visual presentations and written material. Training is continuing as frequent turnover of staff, especially clinicians, is a challenge in The Gambia.

#### Data management and analysis

Nurses complete screening forms for each patient referred to a clinician. Clinicians complete case report forms and investigation request forms. Nurse coordinators take all these forms and specimens to the data management team and laboratory respectively (Figure 2). Specific forms have been created for microbiological analysis and serotyping. Data are double entered and backed up, and paper forms are stored securely. All laboratory and radiological information are fed back by nurse coordinators to those responsible for the patients' clinical management. Formal classification of X-rays is conducted at a later date by trained clinicians blinded to the date of investigation. The data management team produces 3-monthly reports to a steering committee that grants access to the data to investigators and others as requested. The reports include analyses of the main and secondary outcomes and the numbers of suspected and confirmed cases. Formal publication of pre- and post-vaccine introduction data will be in peer-reviewed open access journals.

#### Piloting

After a 1-year preparation period, we established surveillance progressively over a 7-month piloting period. During this time, we screened over 1,000 patients among whom 37 cases of IPD were identified. As a result, minor adjustments were made to the system, including to the screening criteria, forms, and standard operating procedures. Key areas for further training of staff were identified and appropriate training provided. A lack of awareness of the surveillance system among non-surveillance clinic staff was identified and addressed. Formal surveillance began on 12 May 2008.

#### Cost

The capital cost of establishing the system was approximately US$500,000 with annual expenditure of approximately US$1.3 million. While the surveillance system provides clinical and laboratory investigations for those presenting to health centres with suspected IPD, their treatment is undertaken by the government health system.

#### Ethics

While the majority of the activities of the surveillance system are simply enhanced routine care, specific patient consent is obtained for any invasive procedures (such as lung aspiration), for storage of samples for future analysis, and for secure storage of identifiable records.

### Evaluation of the Surveillance System

#### System attributes

Table 4 shows the attributes of the system according to US Centers for Disease Control (CDC) evaluation guidelines [17]. These do not include demonstration of the cost-effectiveness of the system and no formal evaluation of this is planned. Standard operating procedures were written for all activities. Interlocking projects include studies of other pathogens and molecular characterisation of pneumococcal isolates.

**Table 4:** Pneumococcal surveillance system attributes.

Attribute	Comment
**Simplicity**	Clear process for defining and investigating patients.
	Limited number of investigations to be considered.
	Limited sources of information (field, clinic, and laboratory).
	Limited follow-up of cases.
	Standardised data entry processes.
	Little modification of data required prior to reporting.
	Some complexity working within and alongside government systems.
	Standard operating procedures and active management is required to ensure consistent results.
**Flexibility**	Interlocking research projects addressing clinical and laboratory questions are possible.
	Able to accommodate research projects on other pathogens/diseases within the Demographic Surveillance System.
**Data quality**	Completeness is evaluated as a performance indicator and validity through clinical review of diagnosed cases
**Acceptability**	High individual and population level participation in the demographic surveillance system and case ascertainment procedures.
	High level collaboration with government agencies.
	Documentation of patient refusals, form completion, form completeness, reporting rates.
**Sensitivity**	The criteria for investigation may miss cases of pneumococcal disease.
	Investigative tools are likely to miss a proportion of true cases of pneumococcal disease with some indication of this provided by (a) monitoring radiological pneumonia and (b) specific study of blood cultures in all under 5 hospital admissions.
**Predictive value positive**	False positive cases minimised by tight case definitions and laboratory standards. Only a proportion of radiological pneumonia is due to pneumococcus.
**Representativeness**	Population-based surveillance enhances representativeness.
**Timeliness**	Limited only by time to culture positivity and data entry and validation.
**Stability**	Indicators have been established to monitor system performance over time.

Conventional microbiology provides pneumococcal isolates for serotyping. However, blood culture has sensitivity for IPD of less than 50% [18]. While two-thirds or more of lower respiratory tract infections have a normal chest X-ray [3],[19], radiological pneumonia has been a useful primary outcome in efficacy trials [3]. A clinical diagnosis of pneumonia has been useful for estimating the preventable burden of disease [20].

Laboratory-based systems, and hospital record-based reports, are vulnerable to bias from changing practice [21]. The Gambian surveillance system monitors numbers of patients presenting and has standardised clinical, radiological, and laboratory procedures. The microbiological methods have been consistent over time ensuring data comparability between studies in The Gambia. The surveillance area is typical of large areas of sub-Saharan Africa. However, international comparisons need to take into account local characteristics such as HIV prevalence, which is estimated to be less than 2% in The Gambia.

#### Performance indicators

Table 5 shows the performance indicators that were developed and the methods used for measuring them [21]. Vaccine supply and delivery are evaluated as part of the assessment of vaccine effectiveness [22]. We produce regular checklists for equipment, staff, sample transport, and other indicators. We developed clinical, X-ray and laboratory log books to enhance consistency between completed forms and data entry. Regular measurement of these indicators facilitates ongoing quality control and the identification of obstacles to the smooth running of the system. Obstacles identified include turnover of staff, laboratory contamination, misunderstandings between surveillance staff and other staff at the clinics, and the logistics of referral and transport of patients. In addition, external factors, such as flooding in the wet season, can affect performance.

**Table 5:** Performance indicators for the Gambian pneumococcal surveillance system and the methods for measuring them.

Performance Area	Indicator	Method
**Vaccine-specific**	Vaccine supply	Regular checks of vaccine supply log books/information systems
	Vaccine delivery	Random check of cold-chain system
	Vaccine coverage	Biennial surveys of random sample of children with review of health card for vaccine delivery
**Impact on other vaccines**	Other vaccine coverage	Biennial surveys of random sample of children with review of health card for delivery of other vaccines
**Demographic surveillance**	Proportion of the population absent or travelling	Annual review of data from each DSS round to show proportion and trends
**Case Identification**	Proportion of patients with suspected pneumonia, meningitis, and septicaemia that are referred	Regular document review of nursing records
	Completeness of form filling	Manual review of filled forms
	Proportion of all IPD that is identified by the system	Focused study of blood cultures for all those admitted to hospital
	Proportion of those referred for investigation that are adequately investigated	Clinical notes review
**Specimen processing**	Proportion of blood cultures taken prior to administration of antibiotics	History of antibiotic use recorded on clinical form and entered into database; time of sample collection and of administration of antibiotics routinely recorded and entered into database
	Proportion of samples reaching the laboratory within 3 h of collection	Time of receipt recorded on forms, details recorded on database
	Proportion of specimens collected that have results	Review of clinical and laboratory records
	Proportion of blood culture specimens that have no result due to contamination	Monthly review of blood culture results and specific clinical and laboratory investigation of contamination rates above 10% of specimens.
	Proportion of pneumococcal isolates with serotyping results	Review of laboratory records
**Radiology**	Proportion of X-rays of acceptable quality	Reporting of X-rays with quality recorded
**Data management**	Data entry error rate	Data cleaning and verification with monthly reports
	Timeliness	Time recorded for data to become available for reporting

## Conclusions

Our surveillance system has been established to document the direct and indirect impact of PCVs on the incidence of IPD and radiological pneumonia in The Gambia. The system is expected to provide crucial information for immunisation policy and to serve as a model for those introducing routine PCV vaccination in diverse settings. It would be helpful if a similar system could be established in a few key locations globally. Evidence of sustained reduction of IPD and radiological pneumonia due to PCVs is important to justify their introduction and ongoing use. Identification of emerging pneumococcal serotypes may also assist the design of future PCVs. Key features of a robust system include standardised case definitions and criteria for screening, investigation, and reporting. It is also important to identify whether available census information can provide reliable estimates of denominator populations for the calculation of incidence rates, in areas where there is no demographic surveillance system. Over the first 2 years of surveillance, 3,938 suspected cases of pneumococcal disease aged 2–59 months, and 707 aged 5 years and over, were screened in the Basse pneumococcal surveillance system. It is expected that the first detailed results from this project will be peer-reviewed and published in 2012 and that the project will continue until at least 2015.

## Abbreviations

IPD: invasive pneumococcal disease
PCV: pneumococcal conjugate vaccine
WHO: World Health Organization
